# When Brain Beats Behavior: Neuroforecasting Crowdfunding Outcomes

**DOI:** 10.1523/JNEUROSCI.1633-16.2017

**Published:** 2017-09-06

**Authors:** Alexander Genevsky, Carolyn Yoon, Brian Knutson

**Affiliations:** ^1^Rotterdam School of Management, Erasmus University, 3000 DR Rotterdam, The Netherlands,; ^2^Psychology Department, Stanford University, Stanford, California 94305, and; ^3^Stephen M. Ross School of Business, University of Michigan, Ann Arbor, Michigan 48109

**Keywords:** accumbens, fMRI, forecasting, frontal, human, prediction

## Abstract

Although traditional economic and psychological theories imply that individual choice best scales to aggregate choice, primary components of choice reflected in neural activity may support even more generalizable forecasts. Crowdfunding represents a significant and growing platform for funding new and unique projects, causes, and products. To test whether neural activity could forecast market-level crowdfunding outcomes weeks later, 30 human subjects (14 female) decided whether to fund proposed projects described on an Internet crowdfunding website while undergoing scanning with functional magnetic resonance imaging. Although activity in both the nucleus accumbens (NAcc) and medial prefrontal cortex predicted individual choices to fund on a trial-to-trial basis in the neuroimaging sample, only NAcc activity generalized to forecast market funding outcomes weeks later on the Internet. Behavioral measures from the neuroimaging sample, however, did not forecast market funding outcomes. This pattern of associations was replicated in a second study. These findings demonstrate that a subset of the neural predictors of individual choice can generalize to forecast market-level crowdfunding outcomes—even better than choice itself.

**SIGNIFICANCE STATEMENT** Forecasting aggregate behavior with individual neural data has proven elusive; even when successful, neural forecasts have not historically supplanted behavioral forecasts. In the current research, we find that neural responses can forecast market-level choice and outperform behavioral measures in a novel Internet crowdfunding context. Targeted as well as model-free analyses convergently indicated that nucleus accumbens activity can support aggregate forecasts. Beyond providing initial evidence for neuropsychological processes implicated in crowdfunding choices, these findings highlight the ability of neural features to forecast aggregate choice, which could inform applications relevant to business and policy.

## Introduction

Traditional economic and psychological theories (such as revealed preferences and behaviorism) imply that an individual's previous choices should provide the best index of their future choices (Bernheim, 2008). Recent research using techniques capable of resolving deep-brain activity at second-to-second resolution [i.e., functional magnetic resonance imaging (fMRI)] suggest, however, that neural activity might complement behavioral predictions of future choice (Tusche et al., 2010; Genevsky and Knutson, 2015). Although brain activity collected with these methods can predict individual choice, its added value in forecasting choice at the aggregate level of markets remains less clear (Ariely and Berns, 2010). The growing availability of Internet market-level choice data, however, opens new opportunities for researchers to test whether brain activity in an experimental sample can be used to forecast aggregate choice (Berns and Moore, 2012; Dmochowski et al., 2014; Genevsky and Knutson, 2015).

Some components of individual choice might provide more general information about aggregate choice than others. For example, according to an affect–integration–motivation (AIM) framework, ascending neural circuits first affectively evaluate objects, then integrate these evaluations, and then translate evaluations into motivated approach or avoidance (Samanez-Larkin and Knutson, 2015). Even if affective reactions generalize across individuals, value integration may incorporate more-specific multidimensional considerations (e.g., probability, risk, time), which may enhance choice consistency within an individual (i.e., thus “rationalizing” choice; Camille et al., 2011), but paradoxically decrease generalizability across individuals (Kim et al., 2007). Thus, whereas both affective evaluation and value integration might predict individual choice, affective evaluation might more broadly generalize to forecast aggregate choice.

Although neural activity reliably predicts a broad range of individual choices, including purchasing (Knutson et al., 2007; Levy et al., 2011) and financial risk taking (Kuhnen and Knutson, 2005), only a few studies have used neural activity from groups of individuals to forecast aggregate market-level behavior (Falk et al., 2011; Berns and Moore, 2012; Genevsky and Knutson, 2015; Venkatraman et al., 2015; henceforth, “predict” refers to individual choice, while “forecast” refers to aggregate choice). For instance, researchers have used nucleus accumbens (NAcc) activity to forecast aggregate song downloads (Berns and Moore, 2012), but medial prefrontal cortex (MPFC) activity to forecast call volume in response to health-related advertisements (Falk et al., 2011). In these studies, however, researchers did not elicit or compare choice at both individual and aggregate levels of analysis. Thus, researchers have yet to explicitly identify which neural predictors of individual choice generalize to forecast aggregate choice. Here, we sought to use neural activity to both predict individual choice as well as forecast aggregate choice in an Internet crowdfunding market.

The global crowdfunding market is extensive (e.g., having raised over $34.4 billion in 2015; Massolution, 2015) and expanding. Some researchers have begun to explore aspects of crowdfunding transactions, including the influence of personal networks (Mollick, 2014), motivations of project creators (Gerber and Hui, 2013; Belleflamme et al., 2014), and dynamics of project-funding cycles (Agrawal et al., 2013; Kuppuswamy and Bayus, 2015), but researchers have not yet examined motives of individual funders or whether their behavior can be used to forecast aggregate funding success.

Our preliminary goal was to determine whether brain activity in affective circuits predicts individual choices to fund novel crowdfunding projects. Consistent with previous work, we predicted that neural activity in circuits associated with positive arousal (i.e., the NAcc) and value integration (the MPFC) would predict individual choices to fund. Our critical goal, however, was to determine whether neural activity could also forecast crowdfunding outcomes at the aggregate level in an Internet market. Unlike individual choice prediction, but consistent with the AIM framework, we hypothesized that circuits implicated in anticipatory affect (e.g., the NAcc) might forecast market outcomes better than those implicated in value integration (e.g., the MPFC) and possibly even better than individual choice itself. We tested these predictions in a study using fMRI, followed by a replication study designed to verify the findings' generality.

## Materials and Methods

### 

#### 

##### Experimental design and statistical analysis.

In the main and replication studies, pictures and text associated with 36 crowdfunding appeals were presented to 30 subjects, who chose whether to fund each project as they were scanned with fMRI (see *Subjects*, *Crowdfunding task*, and *Project selection*). Subjective ratings of each appeal were then collected immediately after scanning (see *Liking, success, and affect ratings*). For individual choice prediction analyses, fMRI data were preprocessed and extracted from volumes of interest (VOIs) for comparison with behavioral choice and subjective rating predictors (see *fMRI acquisition and analysis*, *Functional connectivity analyses*, and *Classification analyses*). For aggregate forecasting analyses, group-averaged choice, rating, and fMRI VOI data were submitted to classification analyses forecasting eventual Internet funding (or not) of each appeal (see *Classification analyses*).

##### Subjects.

Thirty healthy right-handed human adults participated (14 female; mean age, 23.32 years). Before collecting informed consent, subjects were screened for psychotropic drug use and substance abuse in the past month and for a history of neurological disorders, as well as for typical magnetic resonance exclusions (e.g., metal in the body). None were excluded for excessive head motion (i.e., >2 mm from one scan acquisition to the next). Subjects received $20.00 per hour for participating, plus an endowment of $5.00 cash before scanning for use in the crowdfunding task. All procedures were approved by the institutional review board of the Stanford Medical School. The sample in the replication study was similar, but 35 subjects were recruited and three were excluded for excessive head motion, leaving a total of 32 subjects' data for analysis (17 female; mean age, 23.57 years).

##### Crowdfunding task.

**Figure 1.**
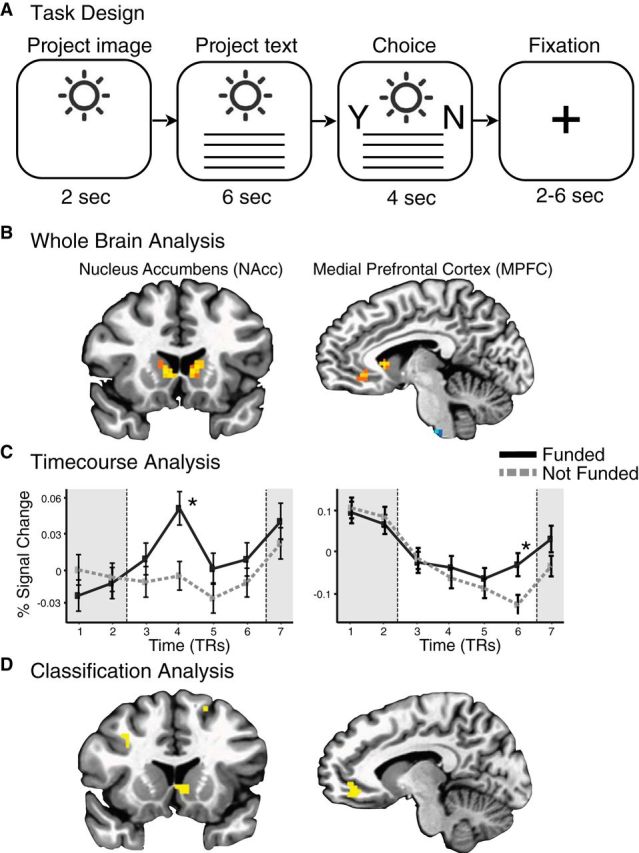
Neural predictors of individual funding choices. ***A***, Neuroimaging task trial design. Subjects saw a project image (2 s), project description (6 s), and spatially counterbalanced prompts to indicate their choice to fund or not (4 s), followed by a variable intertrial fixation interval (2–6 s). ***B***, Whole-brain maps indicating neural activity associated with subjects' choices to fund projects. Warm-colored voxels are positively associated with choices to fund (vs not fund; *p* < 0.05, corrected). Significant clusters of voxels were observed in the bilateral striatum, including the NAcc), as well as in the MPFC. ***C***, Time courses of neural activity extracted from bilateral NAcc (left) and MPFC (right) VOIs during the intertrial interval preceding each trial (TR 1–2; 4 s), project presentation (TR 3–6; 8 s), and choice period (TR 7; 2 s). Separate lines indicate trials in which subjects chose to fund (black, solid) versus not to fund (gray, dashed). Both regions show increased activity while viewing the project associated with subsequent choices to fund (*p < 0.05, corrected). ***D***, Classification of individual funding choices. Whole-brain maps illustrate the top 1% of voxels that predicted individual choices to fund (yellow). As with whole-brain univariate analyses, this model-free classifier identified predictive voxel clusters in the NAcc and MPFC.

Subjects were informed that during scanning, they would make funding decisions regarding a number of actual projects that had been posted on-line on a crowdfunding website (www.kickstarter.com), one of which would be randomly selected after the session. This funding task was therefore incentive compatible and designed to simulate the experience of making on-line crowdfunding choices as closely as possible, while controlling for potential confounds (e.g., related to others' choices and progress toward a funding criterion) and simultaneously facilitating measurement of neural responses to different elements of each funding appeal before choice (Genevsky and Knutson, 2015; Fig. 1*a*). During each funding task trial, subjects first viewed a photographic image from the project page (2 s), followed by a screen depicting the remainder of the project's text description (6 s). Subjects were then asked to indicate whether they would like to fund the project using spatially counterbalanced (i.e., left or right) “Yes” or “No” prompts by pressing one of two corresponding buttons (4 s). After indicating their choice, a colored border highlighted the choice until the choice period ended. Finally, subjects viewed a centrally presented fixation cross (variable 2–6 s) until the beginning of the next trial. Total trial duration (including intertrial interval) thus averaged 16 s (range, 14–18 s).

Subjects encountered a total of 36 funding requests, each of which presented a unique project selected from the crowdfunding website. After scanning, one trial in the funding task was selected at random. If subjects had agreed to fund the randomly selected appeal, that amount was removed from their payment and contributed on-line to the appropriate project; otherwise, subjects retained their full endowment. Subjects were also informed that if their selected project was subsequently funded on the Internet, they would be able to view the associated film once it had been completed. The procedure in the replication study followed the same format.

##### Project selection.

Projects were selected from the most recently posted documentary film projects on the Kickstarter website (www.kickstarter.com) to control for variation between different project categories. The actual Internet outcomes of these projects had not yet occurred at the time of stimulus identification and data collection. Only after the funding windows for all projects had closed were funding outcomes available for collection. Of the 36 selected projects, 18 were eventually funded by groups of Internet contributors, while the remaining 18 did not reach their funding threshold, and so expired at the end of the funding period. Of the 36 selected projects in the replication study, 14 were eventually funded, whereas the remaining 22 were not.

Project stimuli were derived from appeals presented on the kickstarter.com website. Each stimulus included the project's title, its creator's name, a static image designed by the creator, and a text description of the associated film's content. Based upon the depicted images, projects were evenly sampled from three content categories (i.e., face, places, and text). Thus, the focal points of “face” images included an individual or group of people, “place” images featured either an inanimate object or landscapes, and “text” images were primarily composed of text titles. Selected appeals therefore included one of three types of evenly distributed project images (i.e., face, place, or text). Selected appeals in the replication study contained only two types of evenly distributed project images (i.e., “face” or “place”).

##### Liking, success, and affect ratings.

After scanning, subjects rated how much they liked each project and their predicted likelihood that each project would reach its funding threshold (i.e., project campaign success) on seven-point scales (Genevsky and Knutson, 2015). After scanning, subjects also rated their own affective responses to each project proposal using two seven-point scales (one indexing valence from positive to negative and the other indexing arousal from highly arousing to not arousing). Written instructions and spoken clarifications delivered by the experimenter first described the nature of each scale and provided detailed examples (Knutson et al., 2005). While rating projects, subjects indicated their affective responses based on how they previously felt “when presented with this project.” Since positively and negatively aroused affect most closely align with approach and avoidance motivational states (Knutson et al., 2014), as well as activity in relevant neural circuits (Knutson and Greer, 2008; Knutson et al., 2014), valence and arousal ratings were then transformed into positive-arousal and negative-arousal scores by projecting within-subjects mean-deviated valence and arousal scores onto axes rotated 45° [i.e., positive arousal: (arousal/√2) + (valence/√2); negative arousal: (arousal/√2) − (valence/√2); Watson et al., 1999; Knutson et al., 2005]. The rating procedure for the replication study was similar, but since many ratings were highly correlated in the main experiment, subjects only rated their affective responses to each of the stimuli (i.e., with respect to valence and arousal).

##### fMRI acquisition and analyses.

Images were acquired with a 3.0 T General Electric MRI scanner using a 32-channel head coil. Forty-six 2.9-mm-thick slices (in-plane resolution, 2.9 mm cubic; no gap; interleaved acquisition) extended axially from the mid-pons to the crown of the skull, providing whole-brain coverage and good spatial resolution of subcortical regions of interest (e.g., midbrain, NAcc, orbitofrontal cortex). Whole-brain functional scans were acquired with a T2*-weighted gradient echo pulse sequence (TR = 2 s; TE = 24 ms; flip angle, 77°). High-resolution structural scans were acquired with a T1-weighted pulse sequence (TR = 7.2 ms; TE = 2.8 ms; flip angle, 12°) after functional scans, to facilitate their localization and coregistration.

Whole-brain analyses were conducted using Analysis of Functional Neural Images (AFNI) software (Cox, 1996). For preprocessing, voxel time series were sinc interpolated to correct for nonsimultaneous slice acquisition within each volume, concatenated across runs, corrected for motion, slightly spatially smoothed to minimize effects of anatomical variability (FWHM, 4 mm), high-pass filtered (admitting frequencies with period <90 s), and normalized to percentage signal change with respect to each voxel's average over the entire task. Visual inspection of motion-correction estimates confirmed that no subject's head moved >2.0 mm in any dimension from one volume acquisition to the next.

For whole-brain analyses, regression models included eight regressors of no interest (i.e., six indexed residual motion and two indexed activity associated with CSF and white matter intensity; Chang and Glover, 2009). For analysis of sensory input, regressors of interest orthogonally contrasted face versus place stimuli and text versus face and place stimuli. For analysis of individual (i.e., laboratory sample) funding choices, the regressor of interest orthogonally contrasted trials in which subjects chose to fund the projects versus those in which they did not. For neural forecasting analysis of group funding choices on the Internet, the regressor of interest orthogonally contrasted trials in which subjects viewed projects that were later fully funded on the Internet versus those that did not eventually receive funding. Before inclusion in the models, all regressors of interest were convolved with a single γ-variate function that modeled a canonical hemodynamic response (Cohen, 1997). Maps of *t* statistics for the regressor of interest were transformed into *Z* scores, coregistered with structural maps, spatially normalized by warping to Talairach space, and resampled as 2 mm cubic voxels. Group maps were initially voxelwise thresholded (at *p* < 0.005) and then cluster thresholded using a gray matter mask (cluster size, >17 contiguous 3 mm cubic voxels) to yield a corrected threshold for detecting whole-brain activation (*p* < 0.05 corrected). Cluster size was derived via 15,000 Monte Carlo iterations using AFNI program 3dClustSim (version 16.0.06).

Regionally targeted analyses were conducted by specifying VOIs in regions associated with anticipatory affect [NAcc and anterior insula (AIns); Knutson and Greer, 2008] as well as value integration (MPFC; Knutson et al., 2007; Plassmann et al., 2007; Samanez-Larkin and Knutson, 2015) in previously published research. Specifically, spherical VOIs (8 mm diameter) were placed in foci in bilateral value-processing targets in the NAcc (Talairach coordinates: ±10, 12, −2), AIns (±34, 24, −4), amygdala (±24, −5, −15), and MPFC (±4, 45, 0). We further identified VOIs associated with sensory input relevant to project images in regions implicated in processing faces (Kanwisher et al., 1997), places (Epstein and Kanwisher, 1998), and text (Poldrack et al., 1999; Vigneau et al., 2006). Based on independent meta-analytic analyses from the Neurosynth database (http://www.neurosynth.org; Yarkoni et al., 2011), foci for these sensory input VOIs were placed in the fusiform gyrus (FG; ±40, −50 −18), parahippocampal gyrus (PG; ±22, −42, −6), and left inferior frontal gyrus (left IFG; −46, −14, 28). fMRI activity (percentage signal change) was first averaged within each VOI, then averaged across bilateral VOIs, and finally extracted to derive activity time courses.

##### Functional connectivity analyses.

A psychophysiological interaction (PPI) analysis identified context-dependent modulation of functional connectivity between regions implicated in sensory input (i.e., FG, PG, and IFG) and anticipatory affect (i.e., NAcc; Friston et al., 1997; McLaren et al., 2012; Cisler et al., 2014). Activity time courses were first extracted and averaged from bilateral NAcc VOIs and deconvolved using a γ-variate function modeling a canonical hemodynamic response (Cohen, 1997). An interaction time course was then created by multiplying the deconvolved NAcc time course with a vector indicating trial-by-trial funding choices (with +1 and −1, respectively) and then reconvolved with a γ-variate function to account for the hemodynamic response before inclusion in the model (Gitelman et al., 2003). The associated general linear model thus included eight regressors of no interest (six indexed residual motion, and two indexed activity associated with CSF and white matter intensity; Chang and Glover, 2009), in addition to the NAcc VOI time course, a convolved regressor representing individual choices to fund or not, and the PPI of the NAcc VOI time course and individual choices to fund. Voxelwise regression fits were then submitted to group-level *t*-test contrasts to identify correlated activity across individuals. Finally, normalized voxelwise values from these group fits were averaged across sensory input VOIs in the bilateral FG, the bilateral PG, and the left IFG.

##### Classification analyses.

For classification analyses, trial-level data were first randomly divided into training (80%) and testing (20%) sets. Classification models were implemented using logistic regression and the R caret package (Kuhn, 2008). Model selection and parameter optimization were conducted on the training set using repeated 10-fold cross-validation with three repeats such that the training dataset was further randomly subdivided into 10 blocks. Model feature selection and optimization were conducted by training the classifier on 9 of the 10 blocks and testing on the one held-out block. This process iterated over all 10 training blocks, and the entire procedure was repeated three times. Model accuracy was evaluated by applying the resulting final model on the remaining independent 20% of trials in the testing set that had not been used in any phase of model training. To assess model accuracy, 95% confidence intervals (CIs) were constructed around derived estimates and compared with a no-information rate. Reported *p* values represented the proportion of these distributions that exceeded a null hypothetical value of chance prediction (50%).

For classification of individual funding choices, trials involving “yes” and “no” choices were evenly downsampled (i.e., creating a 50–50% split). After downsampling, subjects contributed an average of 25.10 (of 36 total) trials (SD, 8.76; range, 3–36) to the classification analysis. The number of data points that each subject contributed to the classification analyses was not significantly associated with their predictive accuracy (*r* = 0.279, *p* = 0.142). Individual choice classification analyses were conducted on a trial-to-trial basis, and included subjects' self-report ratings of liking, perceived likelihood of success, positive arousal, negative arousal, and brain activity in the VOIs. For the classification models that included brain activity, percentage signal change was first averaged within each VOI, and then averaged bilaterally.

For whole-brain classification analyses, fMRI activity was extracted from each spatially normalized voxel for each of the four brain image volume acquisitions preceding choice on each trial in each subject. Features were selected using recursive feature elimination, such that 5% of remaining voxels with the lowest fits were removed on each iteration until 1% of the total voxels remained (a threshold that demonstrated the highest classification accuracy using the fewest features). Remaining voxel weights were then back-projected into normalized brain space over time to identify where and when features significantly classified funding choice. For whole-brain classification of individual funding choices, accuracy was assessed with leave-one-subject-out cross-validation. On each testing iteration, one subject's data were held out and classified using the model derived from training on the remaining subjects. Accuracies in predicting trial-by-trial choices over 30 subjects were then averaged to predict accuracy in funding choices out-of-sample. Finally, for whole-brain classification of project-level funding outcomes on the Internet, accuracy was assessed using leave-one-project-out cross-validation. On each testing iteration, one project's data were held out and used to assess the accuracy of the model derived from training on the remaining projects. Accuracies in classifying project outcomes over 36 projects were then averaged to generate an overall estimate of accuracy in classifying project outcomes. Forecasts therefore targeted project outcomes (which depended more on funders' choices), rather than amount funded (which depended more on proposers' initial goals).

## Results

### Predicting individual choice

#### Behavioral correlates of individual funding choices

Individual subjects chose on average to fund 14.3 of the 36 presented projects (SD, 5.96; range, 3–27). Similarly, in the replication study, individual subjects chose on average to fund 13.3 of the 36 presented projects (SD, 5.34; range, 2–28). Behavioral analyses first tested associations between individual self-report measures of project liking and funding choices. Independent hierarchical logistic regression models, which included subject as a random effect and predicted trial-to-trial funding choices, indicated that ratings of liking (*z* = 14.57, *p* < 0.001) and perceived likelihood of success (*z* = 11.72, *p* < 0.001) were associated with individual choices to fund. Thus, subjects rated projects that they chose to fund as both more likeable (bootstrapped *t*-test difference estimate, 2.64; 95% CI, [2.48, 2.79]; *t* = 33.04, *p* < 0.001) and more likely to successfully receive their full funding requests (bootstrapped *t*-test difference estimate, 1.12; 95% CI, [0.96, 1.28]; *t* = 13.05, *p* < 0.001). Liking and perceived likelihood of success ratings were then separately averaged across subjects for each project. Bootstrapped correlations (5000 iterations) indicated that ratings of both project liking (*r* = 0.91; 95% CI, [0.83, 0.95]; *p* < 0.001) and perceived likelihood of success (*r* = 0.65; 95% CI, [0.35, 0.84]; *p* < 0.001) correlated with individual choices to fund.

Similar analyses examined associations of self-reported affect ratings with choices to fund. Positive arousal ratings were strongly associated with individual choices to fund (*z* = 13.16, *p* < 0.001), but negative arousal ratings were not (*z* = 0.174, *p* = 0.861). Accordingly, subjects rated projects they chose to fund as evoking more-positive arousal (*t* = 16.25, *p* < 0.001), but not differential negative arousal (*t* = 1.57, *p* = 0.115). Positive-arousal and negative-arousal ratings were then averaged across subjects for each project. A bootstrapped correlation (5000 iterations) indicated that positive arousal ratings for projects correlated with individual funding choices (*r* = 0.61; 95% CI, [0.34, 0.78]; *p* < 0.001). Individual funding choices did not significantly differ, however, as a function of project image type (face, 40%; place, 44%; text, 32%; *F* = 1.09, *p* = 0.35; replication study: face, 42%; place, 36%; *F* = 0.979, *p* = 0.329).

#### Whole-brain predictors of individual funding choices

Whole-brain analyses contrasted brain activity during project presentation (i.e., 8 s) in trials in which subjects subsequently chose to fund versus trials in which they did not. Averaged group brain activity revealed significant clusters that predicted individual choice in the bilateral NAcc and MPFC (Fig. 1*b*).

#### VOI predictors of individual funding choices

**Figure 2.**
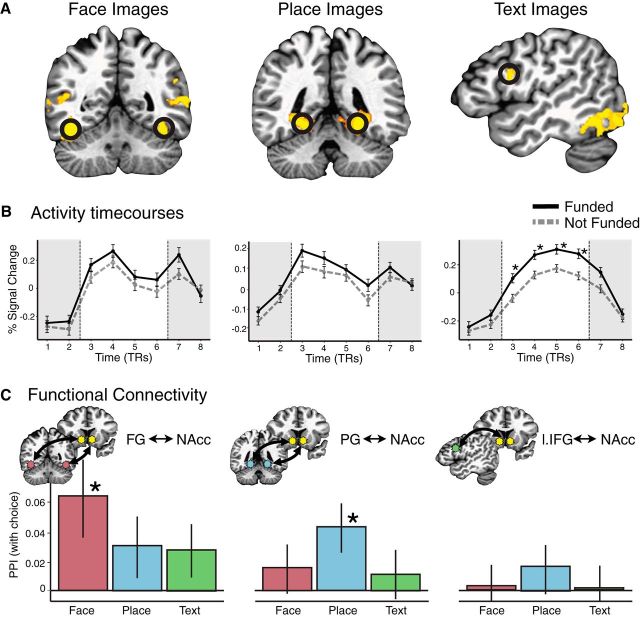
Association of neural activity elicited by project images with individual choices to fund. ***A***, Whole-brain activation maps indicating regions associated with processing project images including face (vs place), place (vs face), and text (vs face + place) stimuli (*p* < 0.05, corrected). Superimposed black circles indicate predefined VOIs based on foci drawn from Neurosynth meta-analyses. ***B***, Activity time courses extracted and averaged over predicted VOIs (***A***). FG (left) and PG (middle) activity did not predict eventual choices to fund (*p < 0.05, corrected). Left IFG (right) activity, however, did predict eventual choices to fund. ***C***, PPIs among activity from FG, PG, IFG, and NAcc VOIs differentially predict choice for stimuli with different image content. Functional connectivity between the FG and NAcc was associated with choice for face stimuli only, while functional connectivity between the PG and NAcc was associated with choice for place stimuli only (*p < 0.05, corrected). Functional connectivity between the left IFG and NAcc, however, was not associated with funding choices in any condition.

Consistent with whole-brain findings, NAcc activity was greater during presentations of projects chosen for funding than for projects not chosen. Activity time course plots (Fig. 1*c*) indicated temporal specificity, with significant differences appearing during the initial part of the project presentation period before subjects could manually indicate their choices. MPFC activity was also greater during presentations of projects chosen for funding than for projects not chosen, but only during the latter part of the presentation period. Consistent with these patterns, a logistic regression indicated that both NAcc (*z* = 2.73, *p* < 0.01) and MPFC (*z* = 2.49, *p* < 0.05) activity at these points significantly and independently predicted trial-by-trial individual choices to fund (Table 1). To address whether sensory processes might also directly contribute to funding choices, a second model incorporated activity from sensory regions (Fig. 2*a*), including the FG, the PG, and the left IFG. Neither the FG (*z* = 0.07, *p* = 0.94) nor the PG (*z* = 1.10, *p* = 0.27) activity predicted choices to fund, but the left IFG activity did (*z* = 3.23, *p* < 0.01; Fig. 2*b*; Table 1). Thus, although a better fit and lower Akaike Information Criterion (AIC) suggested that adding left IFG activity improved predictions of individual choices to fund, this influence did not interact with activity observed in anatomically distinct affective circuits. This pattern of results did not change after controlling for project image type.

**Table 1.** Logistic regressions predicting individuals' trial-by-trial funding choices

	Main study		Replication study	
	Decision VOIs	With input VOIs	Decision VOIs	With input VOIs
NAcc	0.787 (0.261)**^a^	0.723 (0.265)**^a^	0.963 (0.260)**^a^	1.050 (0.266)**^a^
MPFC	0.333 (0.133)*^a^	0.321 (0.135)*^a^	0.476 (0.129)**^a^	0.496 (0.131)**^a^
Insula	−0.178 (0.354)	−0.492 (0.369)	−0.556 (0.362)	−0.557 (0.387)
Amygdala	−0.923 (0.358)*	−1.209 (0.380)*	−0.318 (0.402)	−0.045 (0.433)
FG		0.025 (0.097)		−0.555 (0.215)*
PG		0.202 (0.180)		−0.612 (0.408)
IFG (left)		0.554 (0.164)**		0.845 (0.252)**
Pseudo-*R*^2^	0.142	0.163	0.140	0.158
AIC	1338.0	1323.5	1405.5	1394.4

Statistics are standardized coefficients and SE. Models include fixed effect of stimulus image category.

*^a^*Predicted associations.

*p < 0.05;

**p < 0.01.

#### Functional connectivity

Functional connectivity analysis contrasted correlated activity between the NAcc and the three input-processing region (FG, PG, left IFG) VOIs independently for each of the three project image types (i.e., face, place, and text). A PPI term assessed the degree to which connectivity between these project image regions and the NAcc was associated with individual choices whether to fund projects (Fig. 2*c*). Correlated activity between the NAcc and the FG was significantly associated with individual choices to fund only in the face condition (*t* = 2.136, *p* < 0.05), but not in the place (*t* = 1.547, *p* = 0.133) or text conditions (*t* = 1.726, *p* = 0.100). Similarly, correlated activity between the NAcc and PG was significantly associated with individual choices to fund only in the place condition (*t* = 2.310, *p* < 0.05), but not in the face (*t* = 0.711, *p* = 0.483) or text conditions (*t* = 0.460, *p* = 0.649). Correlated activity between the NAcc and left IFG, however, was not significantly associated with individual choices to fund in any condition (Fig. 2*c*).

#### Classification of individual funding choices

Classification analyses further tested whether different combinations of behavioral and neural data could predict individual funding choices. Logistic regression classifiers were trained on 80% of choice trials (randomly selected) across all subjects and tested on the remaining 20% of trials to classify funded versus unfunded individual choices. Consistent with logistic regression analyses, a first classifier including behavioral self-report ratings of liking, perceived likelihood of success, and affect classified individual funding choices (86.4% accuracy, *p* < 0.001; chance, 50%). A second classifier using neural VOI data alone also significantly predicted individual funding choices (57.8% accuracy; *p* < 0.05). A third classifier combining behavioral and neural data predicted individual funding choices with 85.7% prediction accuracy (*p* < 0.001). A fourth classifier using whole-brain (rather than VOI) neural activity during the project-presentation phase also significantly predicted individual funding choices (58.7%, *p* < 0.05). The amount of data that each individual contributed to classification analyses after even downsampling (see Materials and Methods) was not significantly associated with variation in predictive accuracy (*r* = 0.279, *p* = 0.142).

Whole-brain maps were then reconstructed to visualize selected predictive features in space and time. Consistent with focused univariate predictions, the largest clusters of predictive voxels appeared in the NAcc and MPFC preceding choice (Fig. 1*d*). These features both spatially overlapped with VOIs used in univariate analyses (Fig. 1*b*) and temporally overlapped with periods of discrimination identified in time course activity analyses (Fig. 1*c*). Thus, NAcc features appeared to predict choice before MPFC features, consistent with an account in which anticipatory affect precedes value integration (Samanez-Larkin and Knutson, 2015).

### Forecasting aggregate choice

#### Behavioral forecasts of aggregate choice

Logistic regression analyses next tested whether behavioral and self-report measures from the laboratory sample could forecast aggregate funding outcomes on the Internet, which occurred weeks after the experiment (Table 2). Neither average ratings of project likeability (*z* = −1.171, *p* = 0.242) nor of perceived likelihood of success were associated with Internet funding outcomes (*z* = 0.249, *p* = 0.803). Similarly, average funding choices were also not significantly associated with Internet funding outcomes (*z* = 0.645, *p* = 0.519). Point–biserial correlations specifically verified an absence of significant associations between average ratings of likeability (*r* = −0.231, *p* = 0.879), perceived likelihood of success (*r* = −0.061, *p* = 0.394), and funding choices (*r* = −0.151, *p* = 0.932) with Internet funding outcomes (Table 2). Further, average self-reported affect ratings also did not forecast Internet funding outcomes (Table 2), since both positive arousal ratings (*z* = −1.254, *p* = 0.210) and negative arousal ratings (*z* = 0.279 *p* = 0.780) were not significantly associated with Internet funding outcomes. Image category, however, was associated with Internet funding outcomes (*F* = 6.95, *p* < 0.001), such that appeals depicting face images received more funding (83%) than did those depicting place (17%; *t* = 4.20, *p* < 0.001) or text images (50%; *t* = 1.78, *p* = 0.091, trend). The pattern of reported results did not change, however, after controlling for image category in the models.

**Table 2.** Logistic regressions forecasting aggregate funding outcomes on the Internet for main and replication studies

	Main study				Replication study			
	Behavioral	Affective	Neural	Combined	Behavioral	Affective	Neural	Combined
Funding choice	0.572 (886)			0.761 (1.302)	0.515 (1.632)			1.826 (1.421)
Liking	−1.154 (0.985)			−1.090 (1.564)	—			—
Success likelihood	0.131 (0.528)			0.068 (1.127)	—			—
Positive arousal		−0.489 (0.390)		0.045 (0.749)		−0.729 (0.439)		−3.026 (1.657)^†^
Negative arousal		0.110 (0.392)		0.087 (0.523)		−0.536 (0.405)		−1.337 (0.689)^†^
NAcc			1.691 (0.774)*^a^	1.751 (0.816)*^a^			2.098 (0.940)*^a^	3.872 (2.199)^†^*^a^*
MPFC			−0.991 (0.723)	−0.673 (0.830)			−0.593 (0.509)	−0.557 (0.747)
IFG (left)			−0.729 (0.667)	−0.616 (0.778)			−0.687 (0.457)	−1.217 (0.789)
Amygdala			1.068 (0.702)	0.973 (0.817)			0.126 (0.527)	−0.049 (0.646)
Insula			−0.601 (0.828)	−0.733 (0.932)			−0.665 (0.609)	−0.188 (0.998)
Pseudo-*R*^2^	0.106	0.089	0.236	0.257	0.092	0.183	0.304	0.517
AIC	54.63	53.46	52.14	59.07	49.70	47.28	47.51	43.22
Classification accuracy	52.9	51.8	59.1*	56.5*	55.8	55.2	61.1*	59.3*

Statistics are standardized coefficients and SE. Models include fixed effect of stimulus image category.

a Predicted association.

* *p* < 0.05;

† *p* < 0.10.

#### Neural forecasts of aggregate choice

**Figure 3.**
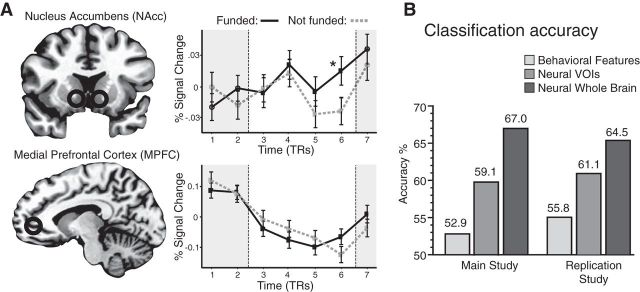
Neural features that forecast Internet funding outcomes. ***A***, VOI activity time courses show that NAcc activity in the laboratory sample significantly classified between projects that were funded (solid black) or not (dashed gray) on the Internet weeks later (*p < 0.05, corrected). MPFC activity, however, did not classify funding outcomes. ***B***, Classification of Internet funding outcomes. Accuracy rates for classification models on main and replication study measures, including behavior and self-report data, neural VOI activity (NAcc) data, and neural whole-brain data.

Activity time courses were extracted from previously identified VOIs (i.e., NAcc, MPFC; see Materials and Methods), as well as VOIs identified in meta-analyses (i.e., left IFG). That is, all activity time courses were extracted from regions based on published anatomical coordinates rather than on current results of individual choice predictions (although coordinates overlapped with those identified in individual choice analyses). Activity in these VOIs were averaged across the laboratory sample for each project, and compared for projects that were either eventually funded or not funded on the Internet (Fig. 3*a*). Averaged time points with significant activation differences were entered into the model predicting funding on the Internet (or all averaged time points, if none significantly differed). During the period preceding choice, only NAcc activity significantly differed for projects that were eventually funded on the Internet versus those that were not. Logistic regression analysis verified that only NAcc activity could forecast Internet funding outcomes (*z* = 2.19, *p* = 0.029; Table 2). Although MPFC and left IFG activity had predicted individual choice in the laboratory sample, activity in these regions did not forecast Internet funding outcomes. Accordingly, the fit of the neural model (pseudo-*R*^2^ = 0.236) exceeded that of either model, including behavioral choice (pseudo *R*^2^ = 0.106) or affect ratings (pseudo-*R*^2^ = 0.089; Table 2). Direct model comparisons indicated that the neural model classified aggregate choice outcomes better than the behavioral model (χ*^2^* deviance = 6.49, *p* = 0.039). Similarly, in the replication study the neural model classified aggregate choice outcomes better than the behavioral model (χ*^2^* deviance = 10.19, *p* = 0.037).

A combined logistic regression model then aimed to forecast Internet funding outcomes by combining behavioral, affective, and neural measures (Table 2). Of these variables, only NAcc activity was significantly associated with Internet funding outcomes (*z* = 2.15, *p* = 0.032). The combined model, however, produced an AIC value greater than the neural model, suggesting that after imposing penalties for additional predictors, the neural model provided a more parsimonious forecast of Internet funding outcomes. To verify that NAcc activity alone could explain significant variance in Internet funding outcomes, we checked independent regression models for activity in each neural region. Consistent with the combined model, only NAcc activity was significantly associated with Internet funding outcomes (*z* = 2.04, *p* = 0.041), whereas both MPFC (*z* = −0.34, *p* = 0.731) and left IFG (*z* = 0.412, *p* = 0.680) activities were not. A permutation test in which NAcc activity was randomly assigned to funded and unfunded trials (across 10,000 iterations) verified that the observed distribution of NAcc activity significantly differed from a randomly constructed null distribution (CI, [0.034, 0.044]; *p* = 0.039).

A second set of logistic regressions applied to data from the replication study yielded similar results. Specifically, behavioral and affective models did not forecast Internet funding outcomes. However, the neural model in general and NAcc activity in particular did forecast Internet funding outcomes, and this effect also trended toward significance in the combined model (Table 2).

#### Classification of aggregate funding outcome

Classification analyses tested the generalizability of the Internet funding forecasts. Logistic regression classifiers were trained on 80% of all projects (randomly selected) and tested on the remaining 20% of projects to classify funded versus unfunded projects. The behavioral model included average ratings of liking, perceived likelihood of success, affect, and funding choices. This behavioral model classified funding outcomes with only 52.9% accuracy, which did not significantly exceed chance (*p* = 0.259), suggesting that behavioral measures of individual choices from the laboratory sample could not forecast Internet funding outcomes. A second targeted neural model then tested whether average VOI activity could classify Internet funding outcomes. This targeted neural model classified Internet funding outcomes with 59.1% accuracy, which exceeded chance (*p* = 0.008), consistent with the notion that neural activity in these regions alone could forecast Internet funding outcomes. A third whole-brain neural model included whole-brain activity during the project-presentation phase of each trial. Cross-validation was achieved by training the model on neural activity from all but one project and then testing on the held-out project. This model classified Internet funding outcomes at 67% (Fig. 3*b*). Replication study classification models yielded similar accuracy rates for the behavioral (accuracy, 55.8%; *p* > 0.05) and neural (accuracy, 61.1%; *p* = 0.002) models (Fig. 3*b*).

Models based only on single-subject VOI data also consistently classified Internet funding above chance (50%; range, 55.5–80.5%; SEM, 1.3%), suggesting that the predictive accuracy of whole-brain classifiers was not driven by outliers, such as a small group of “superforecasters” (Mellers et al., 2015). Maps were reconstructed from the whole-brain model to visualize predictive brain features in space and time. Consistent with regression analyses forecasting Internet funding outcomes, the largest cluster of predictive voxels appeared in the NAcc during the period preceding choice. These features spatially overlapped with those identified in the whole-brain analysis of the laboratory sample (Fig. 1*b*), and temporally overlapped with discriminant activity in time course analyses of Internet funding (Fig. 3*a*).

## Discussion

This research aimed to test whether neural activity could predict individual crowdfunding choices as well as forecast aggregate crowdfunding outcomes on the Internet weeks later. Whereas neural activity in both the NAcc and MPFC predicted individual choices to fund in the laboratory sample, only NAcc activity generalized to forecast aggregate market funding. Further, neural forecasts of market-level outcomes outperformed models that included self-reported ratings of liking, perceived likelihood of success, affective responses, and even individual choices of the laboratory sample. These neural forecasts of aggregate choice were replicated in a second study. Together, the results provide an initial demonstration that a subset of the neural features that predict individual choice can also scale to forecast market-level outcomes.

### 

#### Predicting individual crowdfunding choices

This work makes several novel contributions. First, the findings demonstrate that neural affective measures can predict individual choice in a crowdfunding context, since greater activity in the NAcc and MPFC predicted individual choices to fund. Importantly, this activity occurred before the choice phase of each trial and preceded neural activity associated with the act of indicating a choice. Activity time course analyses also suggested that NAcc activity predicted individual choices to fund before MPFC activity, consistent with accounts like the AIM framework (Samanez-Larkin and Knutson, 2015), which invoke sequential processes of affective evaluation (Knutson et al., 2014) and value integration (Knutson et al., 2007; Plassmann et al., 2007; Levy and Glimcher, 2012). Convergent evidence verified the robustness of these neural predictions, since anatomically targeted regressions, as well as model-free classifiers, implicated both NAcc and MPFC activity in individual choices to fund.

#### Forecasting aggregate crowdfunding outcomes

Second, the findings suggest that some—but not all—features associated with individual choice may scale to forecast aggregate choice at the market level. Sequentially assessing both neural activity and choice in the neuroimaging sample allowed direct comparison of variables that could forecast aggregate choice in an Internet market. Both traditional psychological (i.e., behaviorist) and economic (i.e., revealed preferences) theories imply that behavior in a representative sample of individuals should provide the best forecast of that same behavior at the aggregate level. Thus, if sampled individuals' behavior does not forecast aggregate behavior, then neither should processes that generate that behavior. In the present findings, however, while individual choice in the laboratory could not forecast aggregate behavior, some neural components of choice could.

#### Dissociation from sensory input and motor output

Third, the findings illustrate that decision processes can be distinguished from sensory input and motor output. Presentation of crowdfunding appeals with varying visual content and counterbalanced left versus right motor response requirements allowed dissociation of processes contributing to choice ranging from visual input, to affective evaluation and integration, to motor output. Although the appeals' visual content increased activity in relevant sensory regions (i.e., FG for face stimuli, and PG for place stimuli), these increases did not forecast funding choices. Functional connectivity of activity in these distinct processing regions with NAcc activity, however, did vary as a function of funding choices. Thus, specific images associated with funding requests may have indirectly promoted funding decisions by evoking correlated NAcc activity. These findings suggest that affective activity can flexibly incorporate—but cannot be reduced to—diverse types of sensory input or motor output when supporting choice.

#### Generality of neuroforecasting

While crowdfunding offers an increasingly popular platform for supporting new market ventures, the generalization of these findings to other types of aggregate choice remains unclear. Growing evidence, however, has begun to implicate affective neural activity not only in predicting individual choice, but also in forecasting market outcomes. For instance, research suggests that NAcc activity during passive exposure to novel songs can forecast Internet downloads 2 years later (Berns and Moore, 2012), that NAcc responses during passive exposure to advertisements can forecast advertising-induced increases in sales demand (Venkatraman et al., 2015), and that NAcc responses during exposure to microloan appeals can forecast the success of those appeals on the Internet (Genevsky and Knutson, 2015). While these studies suggest that forecasts from NAcc activity may generalize across diverse market scenarios, only the last study directly compared individual and aggregate choice. Although findings from that study indicated that NAcc activity could add value to forecasts based on affective ratings, they did not demonstrate that brain activity could supplant forecasts based on behavioral data, as we do here. Since most of these Internet markets lack strategic concerns found in traditional financial markets (e.g., auctions, stock trading), future research will need to determine which market conditions are most conducive for application of neuroforecasting (Smith et al., 2014).

The present findings raise the question why both NAcc and MPFC activity predicted individual choice, while only NAcc activity forecasted aggregate choice. Other findings have suggested that MPFC activity can provide information about which antismoking advertisements increase calls to a help line (Falk et al., 2012). NAcc activity may play a more prominent role in choices primarily involving “goods,” but activity in other regions (like the MPFC) may also play roles in choices involving mixtures of “goods” and “bads,” or more complex self-relevant concerns (e.g., including considerations related to probability or time). Future research might systematically explore and manipulate choice scenarios to determine whether and when different neural components support neuroforecasting. The present results provide preliminary support for an account in which affective neural responses generalize more broadly across individuals than processes implicated in value integration.

#### Deconstructing choice to improve forecasts

Conceptually, these findings move beyond accounts that focus solely on choice behavior by seeking to deconstruct processes that underlie choice. The current pattern of results suggests that some components of individual choice might generalize more broadly than others to aggregate choice. This suggests a compromise between accounts in which no individual choices scale to the aggregate versus accounts in which all individual choices scale to the aggregate, by implying that some—but not all—choice components might improve aggregate forecasts. Theory may help to guide further research, since a multistage, hierarchical, neurally situated account of choice (like the AIM framework) counterintuitively but accurately implies that affective components might generalize more broadly than more precise but also more idiosyncratic value integration components. Such evidence may eventually inform applications by indicating that neural activity, in addition to adding value to behavior in aggregate choice forecasts, may also in some cases reveal “hidden information” (Ariely and Berns, 2010). After demonstrating that brain activity can improve aggregate forecasts, investigators' focus may shift toward understanding both the potential and limits of neuroforecasting.

## References

[B1] Agrawal AK, Goldfarb A, Catalini C (2013) Some simple economics of crowdfunding. NBER Working Paper No. w19133. Available at Social Science Research Network (SSRN): https//ssrn.com/abstract=2281044.

[B2] Ariely D, Berns GS (2010) Neuromarketing: the hope and hype of neuroimaging in business. Nat Rev Neurosci 11:284–292. 10.1038/nrn2795 20197790 PMC2875927

[B3] Belleflamme P, Lambert T, Schwienbacher A (2014) Crowdfunding: tapping the right crowd. J Bus Ventur 29:585–609. 10.1016/j.jbusvent.2013.07.003

[B4] Bernheim BD (2008) The psychology and neurobiology of judgment and decision making: What's in it for economists? In: Neuroeconomics: decision making and the brain (Glimcher PW, Fehr E, Camerer C, Poldrack RA, eds), pp 115–125. London: Academic.

[B5] Berns GS, Moore SE (2012) A neural predictor of cultural popularity. J Consum Psychol 22:154–160. 10.1016/j.jcps.2011.05.001

[B6] Camille N, Griffiths CA, Vo K, Fellows LK, Kable JW (2011) Ventromedial frontal lobe damage disrupts value maximization in humans. J Neurosci 31:7527–7532. 10.1523/JNEUROSCI.6527-10.2011 21593337 PMC3122333

[B7] Chang C, Glover GH (2009) Effects of model-based physiological noise correction on default mode network anti-correlations and correlations. Neuroimage 47:1448–1459. 10.1016/j.neuroimage.2009.05.012 19446646 PMC2995588

[B8] Cisler JM, Bush K, Steele JS (2014) A comparison of statistical methods for detecting context-modulated functional connectivity in fMRI. Neuroimage 84:1042–1052. 10.1016/j.neuroimage.2013.09.018 24055504 PMC4019671

[B9] Cohen MS (1997) Parametric analysis of fMRI data using linear systems methods. Neuroimage 6:93–103. 10.1006/nimg.1997.0278 9299383

[B10] Cox RW (1996) AFNI: software for analysis and visualization of functional magnetic resonance neuroimages. Comput Biomed Res 29:162–173. 10.1006/cbmr.1996.0014 8812068

[B11] Dmochowski JP, Bezdek MA, Abelson BP, Johnson JS, Schumacher EH, Parra LC (2014) Audience preferences are predicted by temporal reliability of neural processing. Nat Commun 5:4567. 10.1038/ncomms5567 25072833 PMC4124862

[B12] Epstein R, Kanwisher N (1998) A cortical representation of the local visual environment. Nature 392:598–601. 10.1038/33402 9560155

[B13] Falk EB, Berkman ET, Whalen D, Lieberman MD (2011) Neural activity during health messaging predicts reductions in smoking above and beyond self-report. Health Psychol 30:177–185. 10.1037/a0022259 21261410 PMC3059382

[B14] Falk EB, Berkman ET, Lieberman MD (2012) From neural responses to population behavior: neural focus group predicts population-level media effects. Psychol Sci 23:439–445. 10.1177/0956797611434964 22510393 PMC3725133

[B15] Friston KJ, Buechel C, Fink GR, Morris J, Rolls E, Dolan RJ (1997) Psychophysiological and modulatory interactions in neuroimaging. Neuroimage 6:218–229. 10.1006/nimg.1997.0291 9344826

[B16] Genevsky A, Knutson B (2015) Neural affective mechanisms predict market-level microlending. Psychol Sci 26:1411–1422. 10.1177/0956797615588467 26187248 PMC4570982

[B17] Gerber EM, Hui J (2013) Crowdfunding: motivations and deterrents for participation. ACM Trans Comput Interact 20:32 10.1145/2530540

[B18] Gitelman DR, Penny WD, Ashburner J, Friston KJ (2003) Modeling regional and psychophysiologic interactions in fMRI: the importance of hemodynamic deconvolution. Neuroimage 19:200–207. 10.1016/S1053-8119(03)00058-2 12781739

[B19] Kanwisher N, McDermott J, Chun MM (1997) The fusiform face area: a module in human extrastriate cortex specialized for face perception. J Neurosci 17:4302–4311. 9151747 10.1523/JNEUROSCI.17-11-04302.1997 PMC6573547

[B20] Kim H, Adolphs R, O'Doherty JP, Shimojo S (2007) Temporal isolation of neural processes underlying face preference decisions. Proc Natl Acad Sci U S A 104:18253–18258. 10.1073/pnas.0703101104 17989234 PMC2084329

[B21] Knutson B, Greer SM (2008) Anticipatory affect: neural correlates and consequences for choice. Philos Trans R Soc Lond B Biol Sci 363:3771–3786. 10.1098/rstb.2008.0155 18829428 PMC2607363

[B22] Knutson B, Taylor J, Kaufman M, Peterson R, Glover G (2005) Distributed neural representation of expected value. J Neurosci 25:4806–4812. 10.1523/JNEUROSCI.0642-05.2005 15888656 PMC6724773

[B23] Knutson B, Rick S, Wimmer GE, Prelec D, Loewenstein G (2007) Neural predictors of purchases. Neuron 53:147–156. 10.1016/j.neuron.2006.11.010 17196537 PMC1876732

[B24] Knutson B, Katovich K, Suri G (2014) Inferring affect from fMRI data. Trends Cogn Sci 18:422–428. 10.1016/j.tics.2014.04.006 24835467

[B25] Kuhn M (2008) Building predictive models in R using the caret package. J Stat Softw 28:1–26. 10.1155/2012/94710327774042

[B26] Kuhnen CM, Knutson B (2005) The neural basis of financial risk taking. Neuron 47:763–770. 10.1016/j.neuron.2005.08.008 16129404

[B27] Kuppuswamy V, Bayus BL (2015) Crowdfunding creative ideas: the dynamics of project bakers in Kickstarter. University of North Carolina Kenan-Flagler Research Paper No. 2013-15. Available at Social Science Research Network (SSRN): https://ssrn.com/abstract=2234765 10.2139/ssrn.2234765

[B28] Levy DJ, Glimcher PW (2012) The root of all value: a neural common currency for choice. Curr Opin Neurobiol 22:1027–1038. 10.1016/j.conb.2012.06.001 22766486 PMC4093837

[B29] Levy I, Lazzaro SC, Rutledge RB, Glimcher PW (2011) Choice from non-choice: predicting consumer preferences from blood oxygenation level-dependent signals obtained during passive viewing. J Neurosci 31:118–125. 10.1523/JNEUROSCI.3214-10.2011 21209196 PMC3078717

[B30] Massolution (2015) 2015 Crowdfunding industry report. Available at: http://reports.crowdsourcing.org/index.php?route=product/product&product_id=54.

[B31] McLaren DG, Ries ML, Xu G, Johnson SC (2012) A generalized form of context-dependent psychophysiological interactions (gPPI): a comparison to standard approaches. Neuroimage 61:1277–1286. 10.1016/j.neuroimage.2012.03.068 22484411 PMC3376181

[B32] Mellers B, Stone E, Murray T, Minster A, Rohrbaugh N, Bishop M, Chen E, Baker J, Hou Y, Horowitz M, Ungar L, Tetlock P (2015) Identifying and cultivating superforecasters as a method of improving probabilistic predictions. Perspect Psychol Sci 10:267–281. 10.1177/1745691615577794 25987508

[B33] Mollick E (2014) The dynamics of crowdfunding: an exploratory study. J Bus Ventur 29:1–16. 10.1016/j.jbusvent.2013.06.005

[B34] Plassmann H, O'Doherty J, Rangel A (2007) Orbitofrontal cortex encodes willingness to pay in everyday economic transactions. J Neurosci 27:9984–9988. 10.1523/JNEUROSCI.2131-07.2007 17855612 PMC6672655

[B35] Poldrack RA, Wagner AD, Prull MW, Desmond JE, Glover GH, Gabrieli JD (1999) Functional specialization for semantic and phonological processing in the left inferior prefrontal cortex. Neuroimage 10:15–35. 10.1006/nimg.1999.0441 10385578

[B36] Samanez-Larkin GR, Knutson B (2015) Decision making in the ageing brain: changes in affective and motivational circuits. Nat Rev Neurosci 16:278–289. 10.1038/nrn3917 25873038 PMC5645075

[B37] Smith A, Lohrenz T, King J, Montague PR, Camerer CF (2014) Irrational exuberance and neural crash warning signals during endogenous experimental market bubbles. Proc Natl Acad Sci U S A 111:10503–10508. 10.1073/pnas.1318416111 25002476 PMC4115519

[B38] Tusche A, Bode S, Haynes JD (2010) Neural responses to unattended products predict later consumer choices. J Neurosci 30:8024–8031. 10.1523/JNEUROSCI.0064-10.2010 20534850 PMC6632699

[B39] Venkatraman V, Dimoka A, Pavlou PA, Vo K, Hampton W, Bollinger B, Hershfield H, Ishihara M, Winer RS (2015) Predicting advertising success beyond traditional measures: new insights from neurophysiological methods and market response modeling. J Mark Res 52:436–452. 10.1509/jmr.13.0593

[B40] Vigneau M, Beaucousin V, Hervé PY, Duffau H, Crivello F, Houdé O, Mazoyer B, Tzourio-Mazoyer N (2006) Meta-analyzing left hemisphere language areas: phonology, semantics, and sentence processing. Neuroimage 30:1414–1432. 10.1016/j.neuroimage.2005.11.002 16413796

[B41] Watson D, Wiese D, Vaidya J, Tellegen A (1999) The two general activation systems of affect: structural findings, evolutionary considerations, and psychobiological evidence. J Pers Soc Psychol 76:820–838. 10.1037/0022-3514.76.5.820

[B42] Yarkoni T, Poldrack RA, Nichols TE, Van Essen DC, Wager TD, Van Essen DC, Wager TD (2011) Large-scale automated synthesis of human functional neuroimaging data. Nat Methods 8:665–670. 10.1038/nmeth.1635 21706013 PMC3146590

